# Reproductive performance parameters in a large population of game-ranched white rhinoceroses (*Ceratotherium simum simum*)

**DOI:** 10.1371/journal.pone.0187751

**Published:** 2017-12-13

**Authors:** Cyrillus Ververs, Martine van Zijll Langhout, Miel Hostens, Michelle Otto, Jan Govaere, Barbara Durrant, Ann Van Soom

**Affiliations:** 1 Department of Reproduction, Obstetrics and Herd Health, Faculty of Veterinary Medicine, Ghent University, Salisburylaan, 9820 Merelbeke, Belgium; 2 WildlifeVet.nl, Amsterdam, The Netherlands; 3 Wildlife Vet, Buffalo Dream Ranch, Klerksdorp, South Africa; 4 Institute for conservation research, San Diego Zoo, San Diego, CA, United States of America; Faculty of Animal Sciences and Food Engineering, University of São Paulo, BRAZIL

## Abstract

The population of free-roaming white rhinoceroses (Ceratotherium simum) is under serious threat. Captive breeding of this species is therefore becoming more important, but this is challenging and often not successful. Obtaining reproductive reference values is a crucial aspect of improving these breeding results. In this study performed between 2008 and 2016, reproductive performance was analysed in 1,354 animals kept in a 8000 hectares game-ranched environment. Descriptive statistics of this captive population showed an average annual herd growth (%) of 7 .0±0.1 (min -9 –max 15). Average calving rates were calculated as an annual calving rate of 20% and biennial calving rate of 37% adult females calving per year. Females had a median age of 83.2 months at first calving (IQR 72.9–110.7) and inter-calving intervals of 29.2 (IQR 24.6–34.8) months. Furthermore, translocations of animals did not interfere with reproductive success in terms of inter-calving periods or age at first calving. Multivariate models showed a clear seasonal calving pattern with a significant increase of the number of calvings during December–April when compared to April–December. Our results did not show any significant skewed progeny sex ratios. Weather observations showed no significant influence of rain or season on sex ratios of the calves.

## Introduction

The white rhinoceros (*Ceratotherium simum)* is considered to exist of two subspecies: the Southern white rhinoceros (*Ceratotherium simum simum)*, and the much rarer Northern white rhinoceros (*Ceratotherium simum cottoni)*. The Southern white rhinoceros was exemplary for a major conservation success story. With numbers as low as 50 in the wild in the early 1900s, this subspecies has recently increased to a population of almost 20,000 individuals [1,2]. South Africa represents the major habitat for the Southern white rhinoceros, conserving 16,255 individuals in the wild in 2007 [3]. However, poaching has increased dramatically since 2008, due to a huge demand for rhino horn, and is causing a rapid decline in wild rhinoceros populations and more specifically, of Southern white rhinoceros (SWR) numbers in South Africa. Dwindling population numbers in the wild are mainly due to the inherent value of horn. If the current population numbers continue to diminish at the present rate, with over 1,200 Southern white rhinoceros being poached in South Africa alone in 2015 and over 1,000 in 2016, captive breeding programs may be crucial to prevent extinction. Endeavours to breed white rhinoceros in captivity have led to growth and expansion of population numbers in the past with improved genetic diversity and an increased economic return of investment [4].

Most reports on reproductive performances in zoological institutions have been about individuals or small groups of rhinoceros. Wild-caught southern white rhinoceroses will readily breed in captivity if given appropriate space and food, and if other female rhinos of breeding age are present. However, for reasons that are currently not understood, the rate of reproduction is extremely low among captive-born Southern white females [5]. One of the reasons might be, that in captivity, females are frequently bred for the first time at an older age and that the social hierarchy in smaller groups is often compromised [6,7]. Captive populations kept outside their natural habitat also show a high incidence of prolonged periods of anestrus, with more than half of these females remaining acyclic [7,8] or without ovulation of preovulatory follicles [9–11]. Furthermore, pathologies of the female genital tract, such as endometrial and ovarian cysts, muco- and pyometra, and uterine leiomyoma, adenoma and adenocarcinoma have been documented to occur more in captive rhinoceros than in their free-ranging counterparts [12]. Reproductive pathologies in male rhinoceros have rarely been noticed. Penile edema with prolapse, which prevents normal mating, has been described [12]. Testicular fibrosis (both physiological and pathological), testicular neoplasia and epididymal cysts have been incidentally diagnosed in male rhinoceros at zoological institutions [12–14]. However, reproductive pathologies may be diagnosed more often in captive herds due to more intense management [15]. Adverse physiological effects due to external and internal stimuli, such as diet, metabolism, disease, stress and environment may contribute to a low reproductive success rate in captivity. In fact, only 50% of all captive females reproduce successfully, and only 38% of the females born in captivity have produced offspring [5,16]. In these captive-born a diet-related loss of fertility has been reported as well [17,18]. Interestingly, male-biased sex ratios at birth have long been observed in the captive rhino populations [19].

It is generally accepted that small and isolated populations, like the populations kept in zoos, are vulnerable to stochastic factors [4], and may suffer from biased sex ratios, fluctuation in individual reproductive success [20], and increased inbreeding. The ability to maintain genetic diversity has been studied in detail in wild species, and the effective population size is considered to be 50 to preserve them from short-term genetic risks, but 500 animals are required to maintain long-term adaptability [21]. The same may hold true for reproductive efficiency: many *in situ* and *ex situ* programs for rhinoceros taxa are reporting poor growth rates of the population due to declining reproductive success [22]. Here we postulate that managing small populations of rhinoceros will decrease reproductive efficiency, and that a single meta-population together with large home range size may be necessary for optimal reproduction.

This study analyzed reproductive features in a game-ranched population of SWR with 1,354 individuals that exhibited natural behavior, providing valuable insight into the reproductive performance of confined, game-ranched rhinos. The data generated from this study will hopefully contribute to better understanding and increase breeding success in captive and non-captive white rhinoceroses populations.

## Materials and methods

### Breeding premises and animals

The study animals (n = 1,354) were part of a confined private game-ranched SWR breeding herd and were kept in large breeding camps (9ha per adult animal). The study was conducted with permission of the owner. The game-ranch was located in a semi-arid climate, with high summer temperatures and short, cool, dry winters with frost. The mean annual precipitation was 530 mm, with most rainfall occurring during the summer months. Average midday temperatures for the area ranged from 18°C in June to 29.5°C in January. The region is the coldest during July when the temperature drops to 0°C on average during the night. Access to drinking water was ensured throughout the year. The facility consists of several adjoining properties divided in different breeding camps with 25–70 animals per camp depending on its size and location. To prevent overpopulation, sub-adults (2.5–6 yr) were removed from natal camps and placed in new camps to establish new breeding herds. This allowed population control and prevented inbreeding by creating new gene pools in each breeding population. In addition, new arrival adult bulls were introduced to the herds from outside the facility. Each breeding camp consisted of 1–3 adult breeding bulls with 25–30 adult females, 10–25 calves and 10–25 sub-adults. Each breeding camp was divided into two sections to allow rotational grazing over the summer months, and rhinos were given additional feed when natural grass was limited. Supplementary feed consisted of alfalfa (8 kg) and pellets (5 kg) delivered to each rhino daily during the dry winter season (June-August), then in decreasing amounts until adequate rainfall allowed the return to a fully natural grazing diet. Additional feeding during the winter was designed to maintain the reproductive condition of the animals and assure their general well-being. General additional husbandry requirements, including adequate shelter, shade, mud baths and rubbing post were also available within each campsite. Natural mating occurred without human intervention other than controlling the breeding camp numbers and monitoring the carrying capacity of the field and camp with the minimal surface per rhinoceros set at 9 ha per animal. The rhinoceros were allowed to create their own natural hierarchy and herd structures, as in free-roaming conditions. An ID-microchip was applied in the left neck of each rhinoceroses, and notches were applied in both ears for identification from a distance. A permanent full-time veterinarian was present on the premises for veterinary intervention when needed.

When observed, mounting behavior was recorded. Estrous females showed obvious signs of mounting such as scuff marks on their flanks and back from the legs of the bull. The identity of the bull and the cow were recorded to monitor the fertility of each bull and to predict the possible parturition dates for the cow. Each calving was recorded either early in the morning or late in the afternoon.

The geographical and genetic origin of the animals was diverse, both from free roaming national parks as from private reserves. Animals had been trans-located from different areas, private and national parks into the game-ranch facility. Animals were always darted and fully anesthetized before partially reversed and loaded into crates for transport. During long transport animals were kept tranquilized with Acuphase and azaperone until upon arrival. Females with calf on foot were trans-located in separate crates and reunited upon arrival. Animals were always released straight into a new herd, without any quarantine period. To reduce stress and avoid running through fences, feces found in translocation crates was put in every corner of the new camp. We analysed subsequent calving rates during the first 16 months after translocation to the facility, to determine if translocation affected possible pregnancies. After these 16 months, fertility was no longer linked to translocation.

The game-ranch was conducted in accordance with guidelines presented by the South African Development Community (SADC). The Rhino Program of the SADC aims to maximize the population, ensuring the welfare of the animals as well as long term genetic and demographic diversity. The annual population growth rate reflects the reproductive health of each population and the ability of the individuals in the population to reproduce under each management scheme. This can be defined as a number of key indicators that were used to determine population performance and reproductive health. The analysis reveals factors involved in population performance either above or below the internationally accepted minimum annual growth rate of 5% for rhinoceroses [23]. The calculation of the growth rate excludes translocations in and out of the population during the assessment period.

### Statistics

Data recording started in 2008 when a first group of southern white rhinos arrived at the breeding facility, and continued for 8 years. Data were recorded on Excel spreadsheets (Microsoft, Seattle, WA). All entered data were verified with the original records. Animal data were checked for entry errors and edited if necessary according to the original records. The original data set included 562 lactations of 823 females calving from January 2008 through December 2015. The historical weather data were provided by the South African Weather Service [24] and contained cumulative rain fall (mm) per month from the same time period recorded in the weather station Klerksdorp (26°89´80´´S; 26°62´00´´E at 1329m). Rain data were transformed into 4 categories using quartiles (0.0 –<0.8; 0.8 –<23.6; 23.6 –<52.2; 52.2–182.6 mm rain/month). Rain data were joined with the animal data matching the estimated month of conception.

First, descriptive statistics were calculated to describe the SWR population. These variables included the herd growth, defined as the total number of reproducing animals minus the animals that died, divided by the total number of eligible animals. Eligible animals being defined as adult (≥5 years) animals present plus the number of adult animals that arrived that year. This way annual herd growth is based only on new-born animals within the facility and new arrivals were not counted as herd growth. The effect of the new arrivals on the growth of the population was expressed as the artificial growth parameter, which was calculated as the new arrivals divided by the eligible total of animals at the facility each year. The calving rate (CR) was defined as the percentage of females calving annually and biennially. Given the gestation period of the SWR of ca. 16 months, we found it more interesting to look at biennial calving rates. However, since most previous studies in wild populations refer to annual calving rates we calculated both to make a fair comparison with previous studies. The age at first calving was defined as the number of days between confirmed birth date and first calving date. The inter-calving interval (ICI) was defined as the number of days between two subsequent calvings. On addition to the standard barplots for herd growth and calving rate, scatter- and survival plots were constructed to illustrate, respectively, the variance in age to first calving and effect of translocation on calving. Interquartile ranges (IQR) were calculated as a measure for variability, being equal to the difference between the upper and lower quartiles.

Finally, two multivariate analyses were performed in R (R Development Core Team, 2008) using the *lme4* and *fixed* package to model the number of animals born (family = Poisson) and sex ratio (family = binomial). Generalised mixed effect models were constructed using rainfall quartiles and month of birth nested within the year of birth as fixed effects. Final models were constructed by comparing the Schwarz’s Bayesian information criterion and Akaike’s information criterion (best fit closest to 0). Significance and tendency were declared at *P* < 0.05 and 0.05 < *P* < 0.1 respectively. Data are reported as back transformed reduced model least square means with standard error unless indicated otherwise.

## Results

### Herd growth, calving rate, artificial growth, age at first calving, inter-calving interval and effect of translocation on subsequent calving

The first animals arrived at the facility in 2008. For the 8 years of the study an average of p±36 females was bought annually to expand the population and maintain genetic diversity. The average annual herd growth (%) was 7.0±0.1 (range -9–15). The average artificial growth (%), that includes the number of new arrivals, was 37±32 (range 3–100). Herd numbers increased over the years with new arrivals and calves born (Table 1). Number of calvings between years was also significantly different.

**Table 1:** Population growth parameters from 2008–2015.

Year	2008	2009	2010	2011	2012	2013	2014	2015	Total
Female arrivals	43	70	128	81	101	80	18	36	557
Male arrivals	18	27	19	50	65	49	3	3	234
Number of calvings	2	12	46	82	83	95	117	125	562
Number of deaths	2	27	12	19	14	25	72*	38	209
Cumulative population	61	143	324	519	754	955	1021	1147	1147
Birth/death ratio	1	0.44	3.83	4.37	5.93	3.88	1.63	3.29	
Annual calving rate (%)	4.7	10.6	19	25	20	19	23	23	
Biennial calving rate (%)		15.3	30	45	45	39	42	46	
Herd growth (%)	0	-9	11	15	11	10	6	11	
Artificial growth (%)	100	61	48	30	28	18	3	5	

* In 2014 there was an exceptionally high number of animals that died (72), probably due to exceptional weather conditions (heavy rains and mild floods) and a suspected Clostridium outbreak.

Over 8 years (‘08 –‘15) a total of 562 calvings was recorded within the facility. When the previous calving dates of newly introduced females were available, these data were only incorporated into the sex ratio and seasonality analysis. During the study, 47.26% of the calves born were females and 52.74% were males.

**Fig 1:**
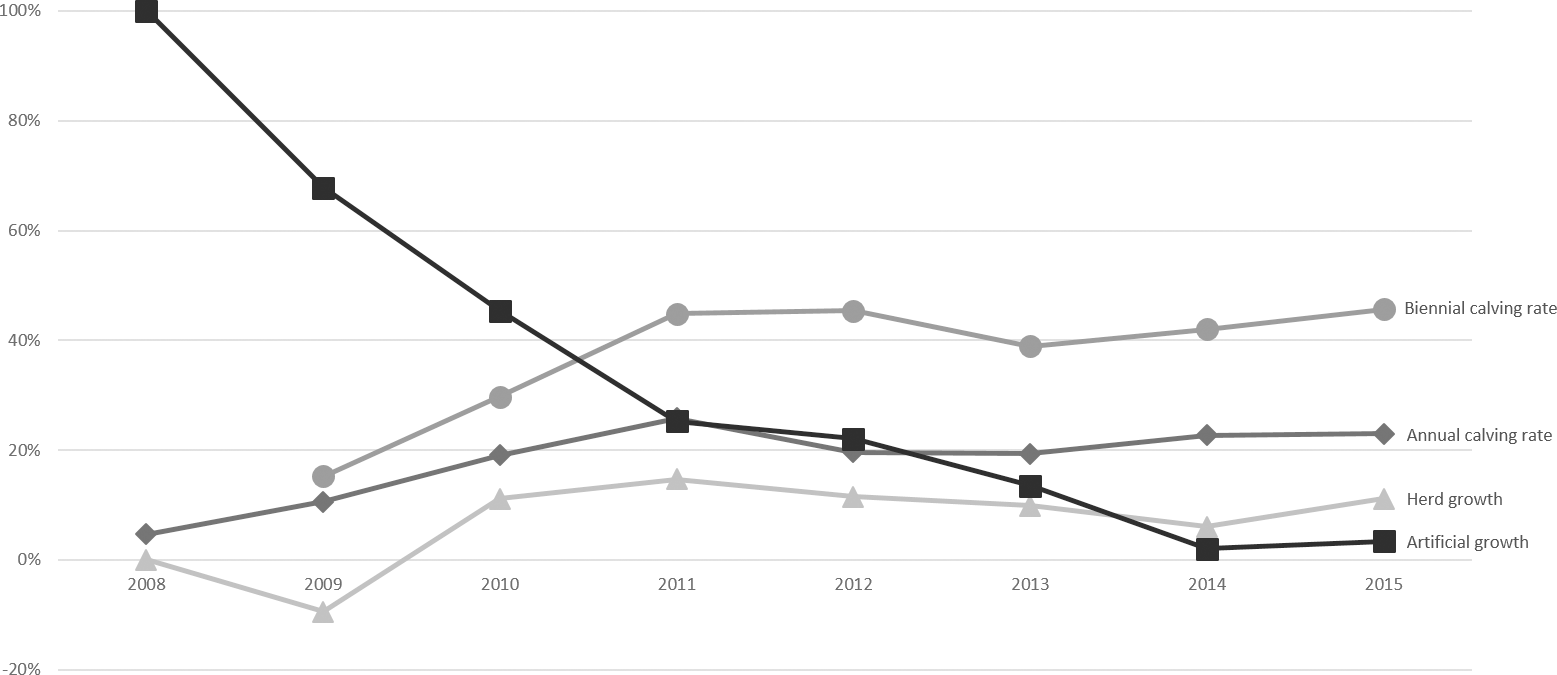
Comparison between the total herd growth, annual and biennial calving rate and the artificial growth. The y-axis shows the total percentage and the x-axis shows the time in years. In 2008 the breeding operation started with new arrivals which explains the 100% of artificial growth.

The average percentage of adult females calving per year (Fig 1) is visualised as the calving rate. This represents the total number of reproducing females divided by the total number of eligible females (the total of eligible females present plus new arrivals and minus females that died). Over the course of the 8 year study an average of 71 ± 46 females each year produced offspring with a minimum of 2 reproducing animals in the first year of the breeding history and gradually increasing to a maximum of 125 reproducing animals out of 588 possible breeding females in 2015. The calving rate was calculated as an annual and biennial rate. The average annual calving rate was 20% and the average biennial calving rate 37%. Since the studied facility expanded each year we also analyzed the artificial growth which was calculated by the number of newly bought animals divided by the eligible total of animals present. Artificial growth did gradually decrease and herd growth gradually increased during the study. Fig 1 shows the evolution of the different parameters that contribute to the population growth at the facility. During the study period only 7 stillbirths were recorded, however, due to extensive farm settings and wild predators the authors decided to include these animals into the dataset without further conclusions regarding total number of stillbirths and abortions. This, then, is a minimum estimate of stillbirths.

**Fig 2:**
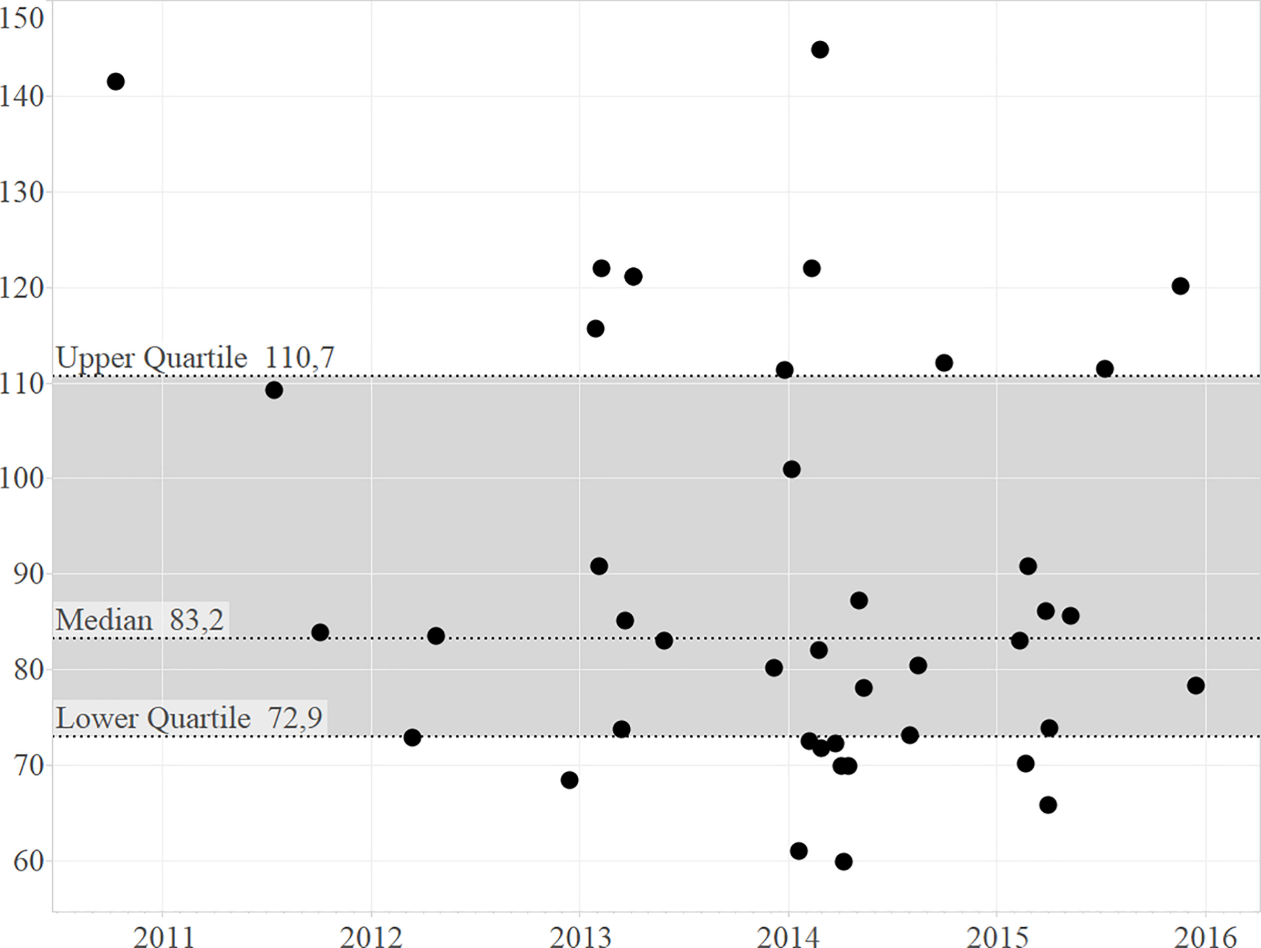
Age at first calving by calving date. The figure shows the age at first calving (month–Y-axis) over time (years–X-axis). The dots in Fig 2 represent nulliparous females that calved.

Fig 2 shows that the median age at first calving was 83.2 months (IQR 72.9–110.7). Records could only be taken from 2009–2016 since only these animals had a documented date of birth and hence a correct age at first calving. Other calving animals only had estimated birth dates as they were bought into the facility. With a gestation period of around 16 months, animals from 2009 only calved at the end of 2010, as shown in Fig 2.

**Fig 3:**
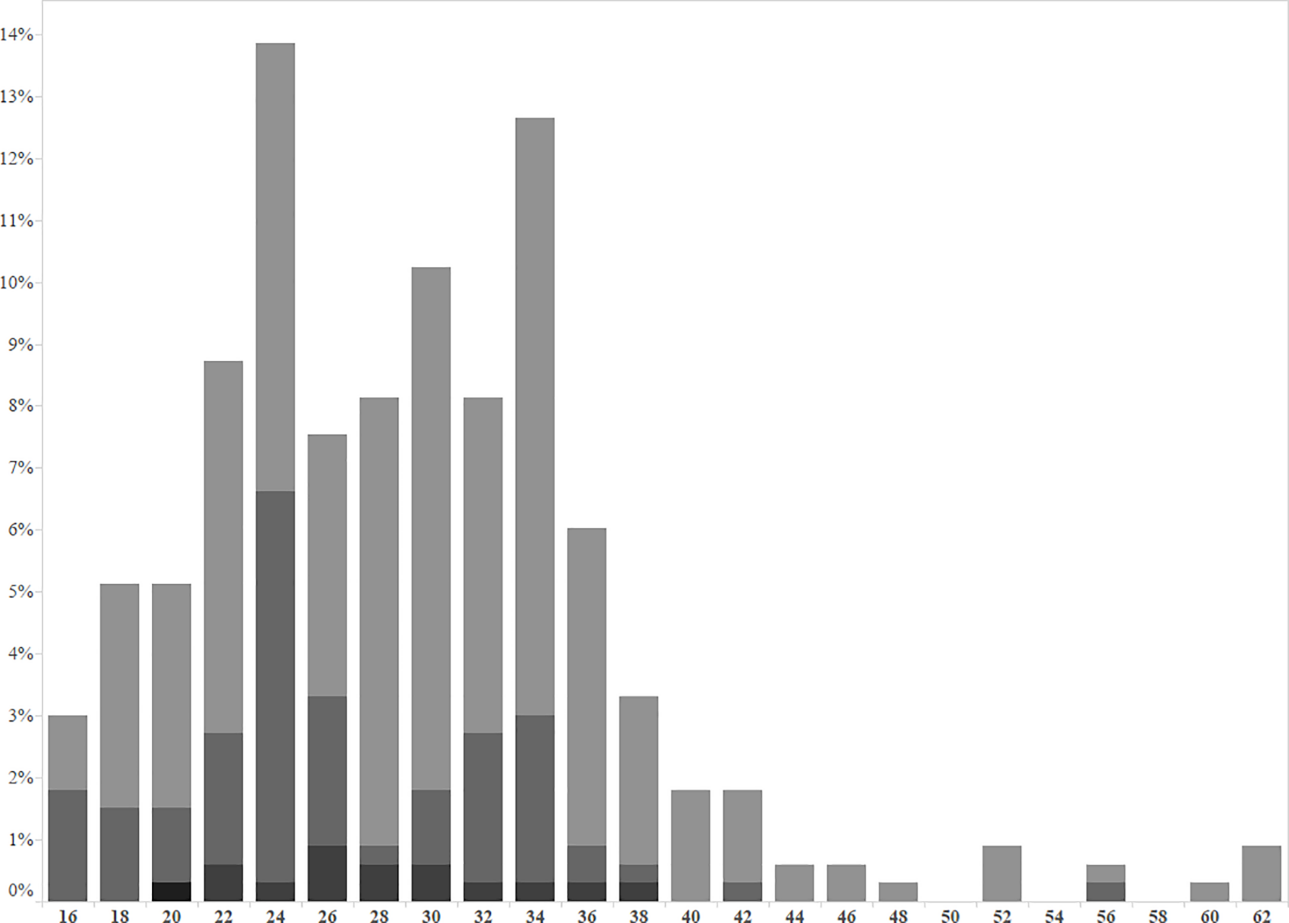
Histogram of calving intervals (in months). The x-axis shows the time interval (months) and the y-axis the percentage of animals. The difference in parity of the animals is indicated through grey scales with lighter bars (parity 1), medium bars (parity 2) and darker bars (parity ≥3) with lower parity animals having lighter bars.

The median overall inter-calving interval in female rhinos of all parities during the study was 29.2 (IQR: 24.6–34.8) months. The inter-calving interval for animals with parity 2, 3 or 4 and more, was 30.8 (IQR: 24.9–35.4), 25.6 (IQR: 23.8–33.1) and 28.0 (IQR: 25.1–32.4) months respectively (Fig 3).

**Fig 4:**
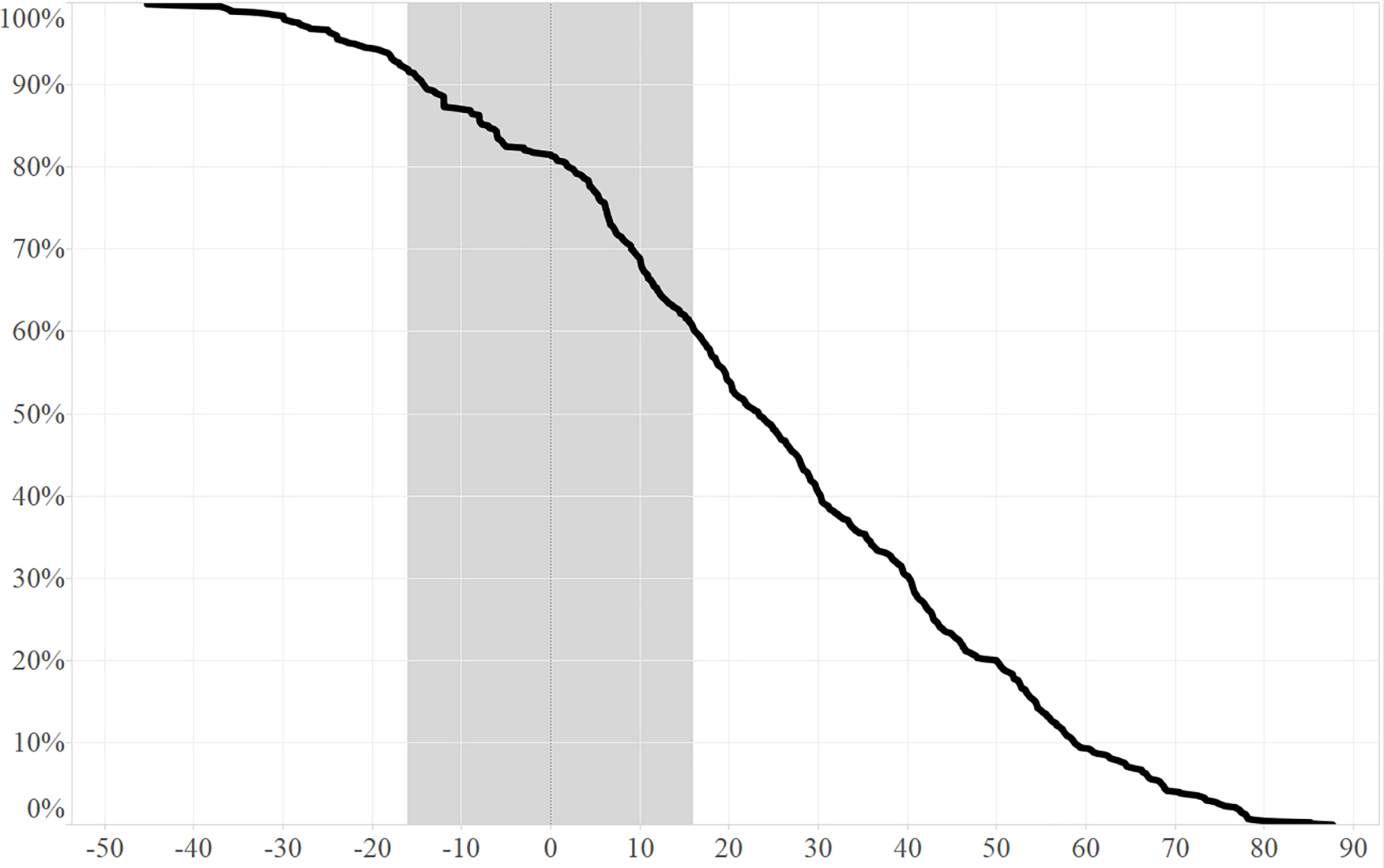
The percentage of animals calving relative to the month of arrival. The x- axis shows the time in months before and after the moment of arrival at the facility. The y-axis indicates the remaining population percentage to calve throughout the study period. The vertical grey bandwidth indicates the ±16 months pre and post arrival interval.

Fig 4 shows the number of months after arrival in relation to the day of first calving at the breeding center. A large percentage of females calved within 16 months of arrival, indicating that these animals were pregnant during translocation. The slope of the graph indicates that the calving rate continues in the same pace before and after translocation. So either the method of translocation was not overly stressful or stress did not affect established pregnancies, as observed abortions were rare in recently translocated females.

By use of a Kaplan-Meier survival analysis we point out that 50% of the arrived females calved within 24 months of the arrival date. With a gestation period of around 16 months, this shows excellent fertility within the total herd. A slight drop in calving rates is seen in the first couple of months before and after arrival (Fig 4).

### Seasonal influences per month on number of births

**Fig 5:**
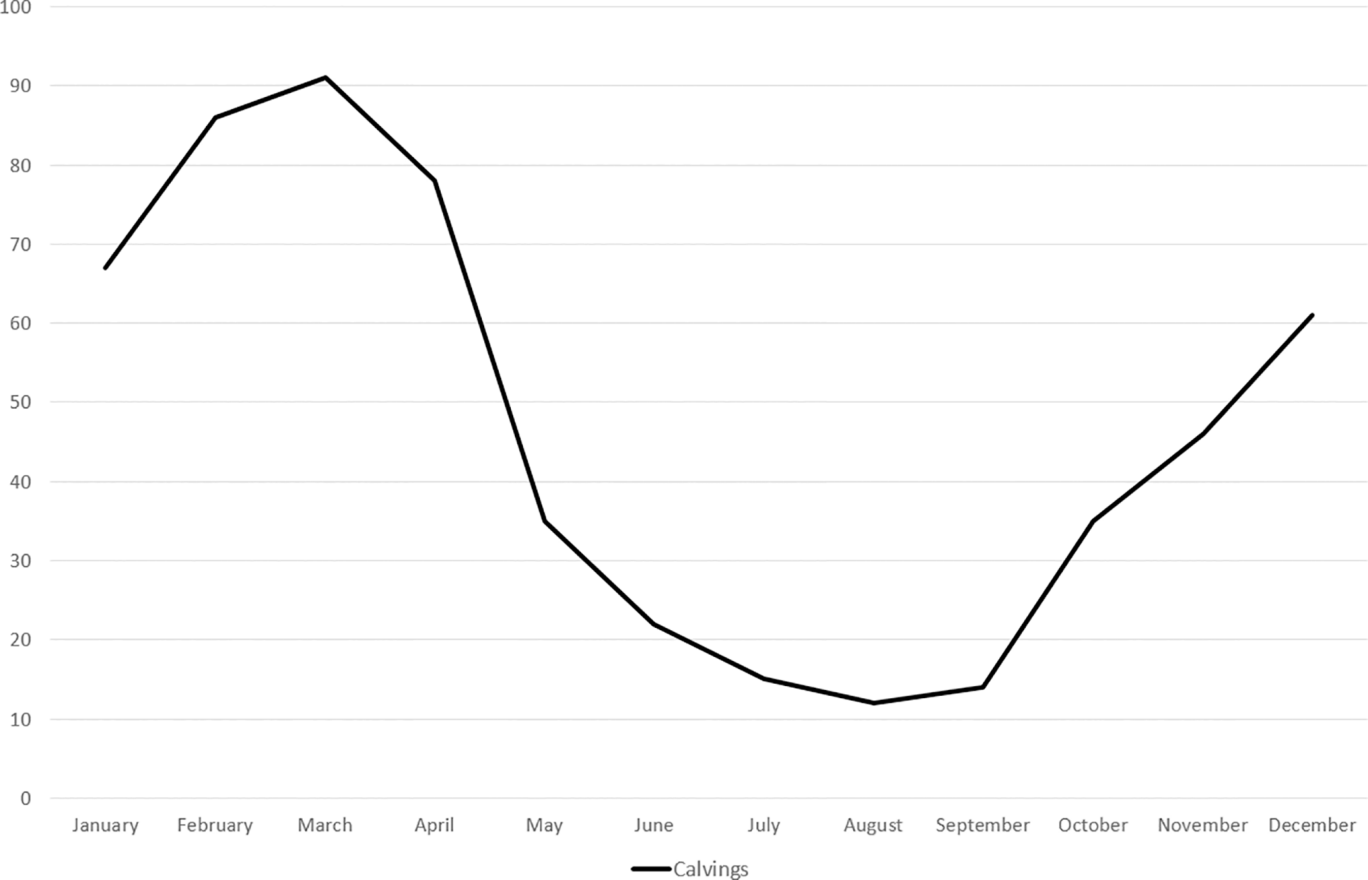
Season of calving. The line shows the total number of calvings throughout the study as divided per month of birth.

The data showed a clear seasonal calving pattern (Fig 5) with a significant increase in number of calvings during December—April in comparison to the rest of the year *(P<0*.*001)*. Fig 5 shows a clear peak in the 562 calvings that took place at the study facility between 2008–2016. With the highest number of births per month being 28 in March 2015.

### Effect of rainfall on number of births and sex ratios

**Fig 6:**
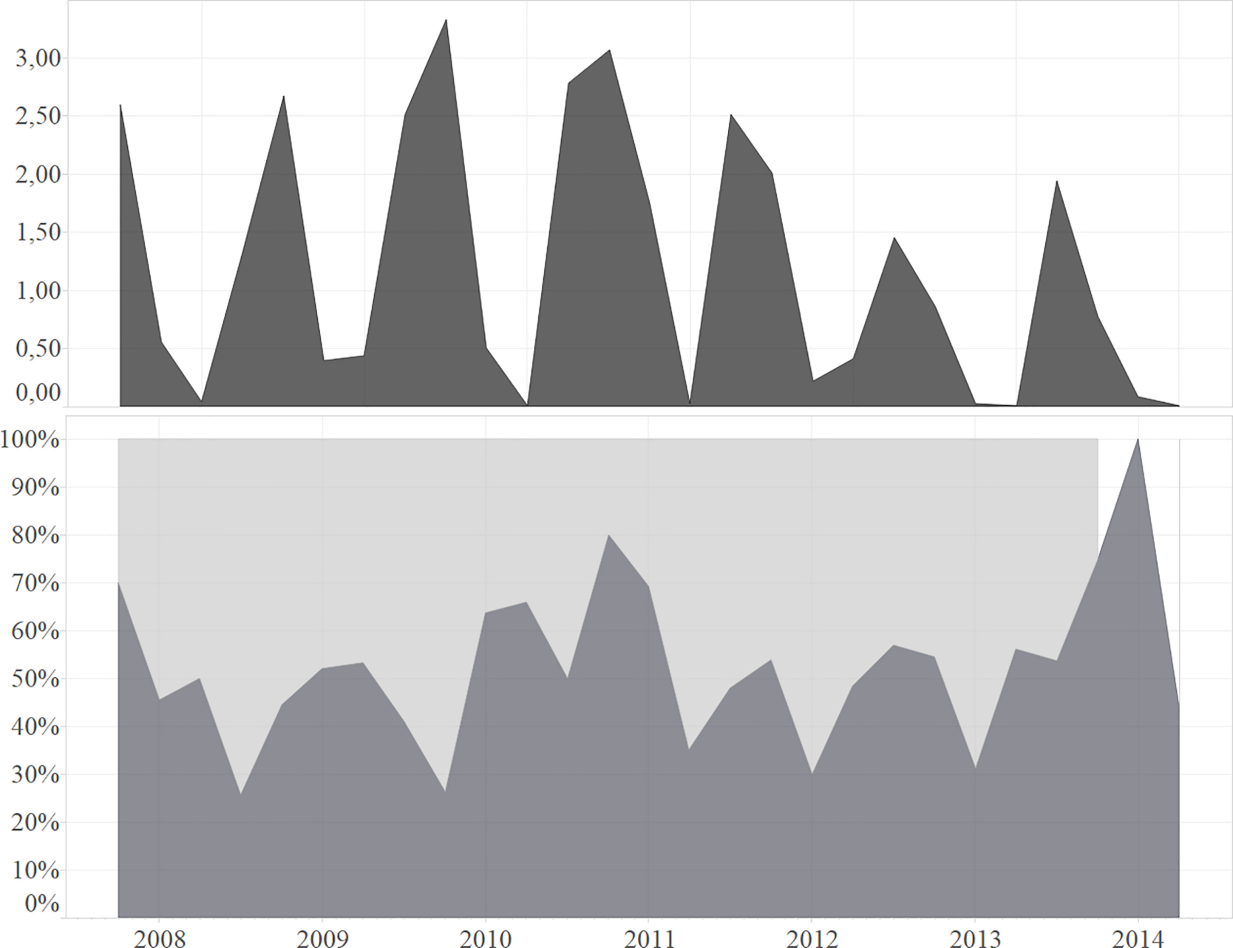
Daily rainfall (upper panel in mm) and percentage of male conceptions (lower panel in %) over time.

Historic rainfall (mm/day) data was determined in the geographical area for 2008–2016 (Fig 6). Throughout the study period, based on the moment of conception, there was no significant effect of rainfall on the calf sex ratio.

## Discussion

In this study, we have analysed for the first time reproductive patterns in a 1,000+ population of game-ranched SWR. Captive breeding has become an important part of species conservation and offspring can be maintained as a genetic reservoir in case they should be needed for reintroduction into the wild or to supplement the existing population with new animals and new genetics [25,26]. Wild SWR populations are decreasing dramatically due to induced death, with over 1,000 poached animals per year in the last three years (environmental affairs, RSA) [27]. The studied population contains more than 5% of the total population left worldwide and is larger than all SWR living in captivity worldwide [20]. This game-ranched breeding institution creates new insights into management, reproduction, genealogy, self-sustainability and animal welfare and it represents a major resource for preservation of genetic diversity.

Reproductive behaviour and performance, as well as expression of appropriate social behaviour are two of the most common obstacles to conservation breeding. Therefore behavioural studies are requested [28–30]. Decreased reproductive performance might be associated with the limited possibilities to exhibit normal reproductive behaviour patterns [5]. In these limited herd sizes from the global captive population, managed in very limited areas and sometimes totally different from the natural conditions of the rhino, the annual growth rate was negative (-3.5%) while growth rates in wild populations were still 6–10% [31]. Crucial to success in captive breeding systems seems to be an appropriate housing and management system, as well as correct diet.

We studied rhinoceros that were kept in large areas roaming at least one dominant male and several adult females. Polygamous breeding, where dominant males have access to several adult females seemed to be required [32–34]. Looking at the key indicators in accordance with guidelines presented by the South African Development Community (SADC) Rhino program, one can conclude that this game-ranch facility had good to excellent reproductive performances [35].

In our study, an average of 20% of the eligible females calved every year and 37% biennially (SADC classifies 33–40% as moderate to good) during the study period (7y). This number is a similar measure of performance to inter-calving interval, however the difference is that the average percentage of adult females calving per year also includes the eligible females that have not calved. Whether one should look at annual or biennial calving rate in animals with a gestation period of more than 12 months is open for discussion. Given the unique set-up of the studied facility, in which total herd growth or population growth was influenced by calves being born at the facility as well as by new animals that were introduced, it is difficult to compare these growth rates with those of previous studies done in wild or captive populations, in which introduction of new animals besides newborn calves is more rare. However, Fig 1 showed that the artificial growth decreased during the study period and the calving rates increased. To avoid misinterpretation of the parameter population growth, we introduced the new parameter herd growth, since this study deals with game-ranched white rhinoceros. Herd growth of game-ranched white rhinoceros is therefore the closest related parameter to compare with population growth in previous studies that looked at wild white rhinoceros. *Rachlow and Berger*, *1998* [36] showed a population growth between 6.6% and 10% in the wild. A population growth of 10.5% had previously been documented as a theoretical maximum for the species [37]. In our study the herd growth was on average 7%, which was mainly caused by the slow start of the breeding operation where population growth was mainly due to new arrivals. With a median age at first calving of 7 years of age (83.21 months), the breeding herd showed results in accordance with rapidly growing populations where females may have their first calves as young as at 6.5 years of age [38]. Other studies showed age at first calving to be 5.6–8 years [39] and 7.4–10.1 years [36,40]. During the course of this study, the age at first calving did not increase significantly when numbers and density of animals increased. This is in contrast with observations in previous studies where a high-density population was compared to a low-density population [36]

Median inter-calving interval of 29.25 month (with < 30 months classified as good to excellent) is even an overestimation since some animals brought in with an incomplete calving history, exhibited a longer inter-calving interval. Animals with higher parity still showed regular inter-calving intervals of 25–27 months intervals, despite regularly anaesthetic procedures for management purposes. In previous studies free roaming rhinoceros showed a 30 months average inter-calving interval [38], or even of 34.8 (low density) to 39.6 months [36], whereas captive white rhinoceros showed a larger variation in inter-calving period from 19.5–29.5 months (91 births from 30 females)[32] or 34 months out of 33 calving intervals calculated of different parity animals [40].

In this study we also looked at translocation of animals when they arrived at the facility and the number of days till first calving at the facility. A slight drop in calving rates is seen in the first couple of months before and after arrival. This is probably because highly pregnant animals and freshly calved animals (calf on foot < 5 months) are very seldom translocated from one facility to another. Other than that the translocation did not seem to affect the general fertility of the animals. However we did not have a control group of the same animals that calved without being translocated but only a follow-up in time of the same population. If we examine the survival analysis of the calvings of the animals that have been translocated into the population, we see the same slope in the first 60 months after calving. This shows that in a time period double the calving interval, there is no change in calving rate. In other words, fertility does not seem to be influenced by stress due to translocation. Even more, management manipulations that necessitate darting (anesthesia) performed on a regular basis, had no influences on fertility.

Our data showed a clear seasonal calving pattern with increased numbers from December until April. Most studies describe reproduction with seasonal peaks or as not seasonally restricted [40,41] but never with such a clear seasonal influence as shown in our data. In this study conception rates were higher during periods of supplemental feeding, therefore further research into nutritional influences of captive and wild diets on reproduction would be of great value. Previous studies pointed out that phytoestrogens might play a negative role in the reproductive success of captive white rhinoceros [17,18,42,43].

Out of 562 new-borns in the facility during the study, there was no significant skewed progeny sex ratio detected. The fact that the dam will adjust the sex of the offspring in response to environmental conditions in order to produce the male sex that has the greatest potential to reproduce, as described for the first time in the hypothesis of Trivers and Willard (1973), is currently observed in most captive breeding facilities [4,44]. This skewed sex ratio could ultimately result in further reducing the rhinoceros population reproductive success in future with bulls outnumbering cows. In this case, removal of males from small populations might be indicated for long term survival of the population. When we modelled the rainfall during the time of conception, then it showed no significant influence on the sex of the offspring, with an overall progeny sex ratio of 53.1% males.

Based on reproductive performances, we can conclude that it is possible to breed a large number of rhinoceros in game-ranched conditions that allowed exhibition of normal social behaviour, with proper implementation and control of animal welfare, habitat and management. Captive as well as wild reproductive performances were exceeded. Populations as studied might be of great genetic value for future genetic variety of free roaming populations and can help fight the losing battle against poaching.

## References

[1] Amin R, Thomas K, Emslie R, Foose T, Strien N (2006) An overview of the conservation status of and threats to rhinoceros species in the wild. International Zoo Yearbook 40: 96–117.

[2] Emslie R, Milliken T, Talukdar B (2012) African and Asian rhinoceroses—status, conservation and trade: A report from the IUCN Species Survival Commission (IUCN/SSC) African and Asian Rhino Specialist Groups and TRAFFIC to the CITES Secretariat pursuant to Resolution Conf. 9.14 (Rev. CoP15). CITES: CoP16 Doc. 54.2 (Rev. 1). CITES: CoP16 Doc 542 (Rev 1).

[3] Ripple WJ, Newsome TM, Wolf C, Dirzo R, Everatt KT, Galetti M, et al (2015) Collapse of the world’s largest herbivores. Science Advances 1: e1400103 doi: 10.1126/sciadv.1400103 26601172 10.1126/sciadv.1400103 PMC4640652

[4] Garnier JN, Bruford MW, Goossens B (2001) Mating system and reproductive skew in the black rhinoceros. Molecular Ecology 10: 2031–2041. 11555246 10.1046/j.0962-1083.2001.01338.x

[5] Swaisgood RR, Dickman DM, White AM (2006) A captive population in crisis: testing hypotheses for reproductive failure in captive-born southern white rhinoceros females. Biological Conservation 129: 468–476.

[6] Metrione LC, Penfold LM, Waring GH (2007) Social and spatial relationships in captive southern white rhinoceros (Ceratotherium simum simum). Zoo biology 26: 487–502. doi: 10.1002/zoo.20143 19360596 10.1002/zoo.20143

[7] Hermes R, Hildebrandt TB, Walzer C, Goritz F, Patton ML, Silinski S, et al (2006) The effect of long non-reproductive periods on the genital health in captive female white rhinoceroses (Ceratotherium simum simum, Ceratotherium simum cottoni). Theriogenology 65: 1492–1515. doi: 10.1016/j.theriogenology.2005.09.002 16213012 10.1016/j.theriogenology.2005.09.002

[8] Hermes R, Göritz F, Streich WJ, Hildebrandt TB (2007) Assisted reproduction in female rhinoceros and elephants–current status and future perspective. Reproduction in Domestic Animals 42: 33–44. doi: 10.1111/j.1439-0531.2007.00924.x 17688600 10.1111/j.1439-0531.2007.00924.x

[9] Radcliffe RW, Czekala NM, Osofsky SA (1997) Combined serial ultrasonography and fecal progestin analysis for reproductive evaluation of the female white rhinoceros (Ceratotherium simum simum): preliminary results. Zoo Biology 16: 445–456.

[10] Roth TL, Bateman HL, Kroll JL, Steinetz BG, Reinhart PR (2004) Endocrine and ultrasonographic characterization of a successful pregnancy in a Sumatran rhinoceros (Dicerorhinus sumatrensis) supplemented with a synthetic progestin. Zoo Biology 23: 219–238.

[11] Stoops MA, Pairan RD, Roth TL (2004) Follicular, endocrine and behavioural dynamics of the Indian rhinoceros (Rhinoceros unicornis) oestrous cycle. Reproduction 128: 843–856. doi: 10.1530/rep.1.00328 15579602 10.1530/rep.1.00328

[12] Hermes R, Hildebrandt TB (2011) Rhinoceros theriogenology In: Miller R.E., Fowler M.E. (Editors) Fowler´s Zoo and Wild Animal Medicine Current Therapy: Elsevier Health Sciences, 546–561. 546–561 p.

[13] Portas TJ, Hermes R, Bryant BR, Göritz F, Ladds P, Hildebrant TB (2005) Seminoma in a southern white rhinoceros (Ceratotherium simum simum). Veterinary Record 157: 556–558. 16258138 10.1136/vr.157.18.556

[14] Portas TJ, Hildebrandt TB, Bryant BR, Göritz F, Hermes R (2010) Seminoma in a southern black rhinoceros (Diceros bicornis minor): diagnosis, surgical management and effect on fertility. Australian Veterinary Journal 88: 57–60. doi: 10.1111/j.1751-0813.2009.00532.x 20148829 10.1111/j.1751-0813.2009.00532.x

[15] Ververs C, van Zijl Langhout M, Govaere J, Van Soom A (2015) Features of reproduction and assisted reproduction in the white (Ceratotherium simum) and black (Diceros bicornis) rhinoceros. Vlaams Diergeneeskundig Tijdschrift 84: 175–187.

[16] Metrione LC, Harder JD (2011) Fecal corticosterone concentrations and reproductive success in captive female southern white rhinoceros. General and comparative endocrinology 171: 283–292. doi: 10.1016/j.ygcen.2011.02.010 21354160 10.1016/j.ygcen.2011.02.010

[17] Tubbs CW, Moley LA, Ivy JA, Metrione LC, LaClaire S, Felton RG, et al (2016) Estrogenicity of captive southern white rhinoceros diets and their association with fertility. General and comparative endocrinology. 10.1016/j.ygcen.2016.05.004 27167501

[18] Tubbs C, Hartig P, Cardon M, Varga N, Milnes M (2012) Activation of southern white rhinoceros (Ceratotherium simum simum) estrogen receptors by phytoestrogens: potential role in the reproductive failure of captive-born females? Endocrinology 153: 1444–1452. doi: 10.1210/en.2011-1962 22253418 10.1210/en.2011-1962 PMC3281539

[19] Dennis PM, Rajala-Schultz PJ, Funk JA, Blumer ES, Miller RE, Wittum TE, et al (2007) Risk factors associated with a skewed natal sex ratio in captive black rhinoceroses (Diceros bicornis) in the United States. Journal of Zoo and Wildlife Medicine 38: 533–539. doi: 10.1638/MS05-011.1 18229858 10.1638/MS05-011.1

[20] Foose T, Wiese R (2006) Population management of rhinoceros in captivity. International Zoo Yearbook 40: 174–196.

[21] Soulé ME (1980) Thresholds for survival: maintaining fitness and evolutionary potential. Conservation biology: an evolutionary-ecological perspective 111: 124.

[22] Mills A, Morkel P, Amiyo A, Runyoro V, Borner M, Thirgood S (2006) Managing small populations in practice: black rhino Diceros bicornis michaeli in the Ngorongoro Crater, Tanzania. Oryx 40: 319–323.

[23] Du Toit J (1998) Rhino Ranching: A Manual for Owners of White Rhinos: Africa Publishers.

[24] (SAWS) SAWS (2008–2016).

[25] Magdalena Wolf C, Garland T Jr, Griffith B (1998) Predictors of avian and mammalian translocation success: reanalysis with phylogenetically independent contrasts. Biological conservation 86: 243–255.

[26] Fischer J, Lindenmayer D (2000) An assessment of the published results of animal relocations. Biological conservation 96: 1–11.

[27] international str (2016) Rhino population figures.

[28] Wielebnowski N (1998) Contributions of behavioral studies to captive management and breeding of rare and endangered mammals. Behavioral ecology and conservation biology: 130–162.

[29] Lindburg DG, Fitch-Snyder H (1994) Use of behavior to evaluate reproductive problems in captive mammals. Zoo Biology 13: 433–445.

[30] Swaisgood RR (2007) Current status and future directions of applied behavioral research for animal welfare and conservation. Applied Animal Behaviour Science 102: 139–162.

[31] Emslie R, Brooks M (1999) African rhino: status survey and conservation action plan: IUCN.

[32] Lindemann H (1982) African rhinoceroses in captivity. The White Rhinoceros Ceratotherium simum (Burchell, 1817) The Black Rhinoceros Diceros bicornis (Linnaeus, 1758) MSc Thesis, University of Copenhagen.

[33] Fouraker M, Wagener T, Emery H (1996) AZA rhinoceros husbandry resource manual.

[34] Owen-Smith RN (1975) The Social Ethology of the White Rhinoceros Ceratotberium simum (Burchell 1817*). Zeitschrift für Tierpsychologie 38: 337–384.

[35] Du Toit R (2006) Guidelines for implementing SADC rhino conservation strategies: WWF-SARPO.

[36] Rachlow JL, Berger J (1998) Reproduction and population density: trade-offs for the conservation of rhinos in situ. Animal Conservation 1: 101–106.

[37] Owen-Smith RN (1992) Megaherbivores: the influence of very large body size on ecology: Cambridge university press.

[38] Owen-Smith RN, Smith RNO (1973) The behavioural ecology of the white rhinoceros: University of Wisconsin Madison.

[39] Patton ML, Swaisgood RR, Czekala NM, White AM, Fetter GA, Montagne JP, et al (1999) Reproductive cycle length and pregnancy in the southern white rhinoceros (Ceratotherium simum simum) as determined by fecal pregnane analysis and observations of mating behavior. Zoo Biology 18: 111–127.

[40] Skinner J, Dott H, Matthee A, Hunt L (2006) Captive breeding of the white rhinoceros, Ceratotherium simum, and the Cape buffalo, Syncerus caffer: research communication. Onderstepoort Journal of Veterinary Research 73: p. 237–239. 17058447

[41] Owen-Smith N (1971) Territoriality in the white rhinoceros (Ceratotherium simum) Burchell. Nature 231: 294–296. 4930978 10.1038/231294a0

[42] Patisaul HB (2012) Infertility in the Southern White Rhino: is diet the source of the problem?: Oxford University Press. 10.1210/en.2012-1008 PMC3320249 22408178

[43] Patisaul HB (2013) Effects of environmental endocrine disruptors and phytoestrogens on the kisspeptin system Kisspeptin Signaling in Reproductive Biology: Springer pp. 455–479. 10.1007/978-1-4614-6199-9_21 23550019

[44] Trivers RL, Willard DE (1973) Natural selection of parental ability to vary the sex ratio of offspring. Science 179: 90–92. 4682135 10.1126/science.179.4068.90

